# Analgesic and Neuroprotective Roles of Dexmedetomidine in Spine Surgery: A Systematic Review

**DOI:** 10.3390/diseases13070212

**Published:** 2025-07-06

**Authors:** Spyridoula Roberta Afrati, Ioanna Lianou, Angelos Kaspiris, Vasileios Marougklianis, Anastasia Kotanidou, Spiros G. Pneumaticos

**Affiliations:** 1 Third Department of Orthopaedic Surgery, School of Medicine, National and Kapodistrian University of Athens, KAT General Hospital of Athens, Nikis 2, 14561 Athens, Greece; roafrati@yahoo.gr (S.R.A.); billmarou@gmail.com (V.M.); spirospneumaticos@gmail.com (S.G.P.); 2 Department of Orthopaedics, “Agios Andreas” General Hospital of Patras-NHS, 262224 Patras, Greece; jolianou@hotmail.com; 3 First Department of Critical Care Medicine & Pulmonary Services, GP Livanos and M Simou Laboratories, National and Kapodistrian University of Athens, Evangelismos Hospital, 10676 Athens, Greece; akotanid@med.uoa.gr

**Keywords:** dexmedetomidine, delirium, pain, analgesics, opioids

## Abstract

Objectives: The incidence of postoperative pain in patients that undergo spinal interventions is significantly increased, affecting their functional outcomes and quality of life. Dexmedetomidine (DEX) belongs to the category of centrally acting nonopioid agents with highly selective α2 adrenoreceptor agonist activity that are frequently applied in spinal surgery based on its antinociceptive and anxiolytic properties. Although many studies displayed the effectiveness of DEX in postoperative pain management, the impact of DEX on functional improvement after spinal surgeries is still debatable. Purpose: This systematic review focuses on the intraoperative and postoperative role of dexmedetomidine (DEX) as an analgesic agent in elective and emergency adult spine surgery. Methods: An electronic literature review search was conducted via Web of Science and PubMed to assess the impact of DEX on postoperative pain management, postoperative delirium (POD), and postoperative cognitive dysfunction (POCD). Discussion: Twenty-one studies were retrieved, three of which were review articles. The effects of DEX were studied for up to 48 h postoperatively. In most cases, its administration was associated with reduced intraoperative and postoperative opioid consumption. However, findings on pain control were less conclusive due to heterogeneity in dosing protocols, concomitant medications, the timing of administration, and pain scoring systems. DEX appears to reduce the incidence of POD and POCD, particularly when used in combination with other drugs. Conclusions: Although the present study supports that the intraoperative administration of dexmedetomidine decreases the pain intensity and/or opioid consumption as well as the development of POD and POCD in patients undergoing spinal surgeries during the first 24 h postoperatively, the current literature should be expanded to allow for the safe generalisation of findings over longer follow-up periods. Further research into the neuroprotective, analgesic, and anti-inflammatory roles of DEX is warranted.

## 1. Introduction

The range of spinal surgeries performed in patients with significant comorbidities, including heart failure, respiratory disease, and vascular conditions, poses considerable challenges for both anaesthesia and surgical teams. Perioperative and postoperative pain management significantly affects patients’ haemodynamic stability, rehabilitation, complication rates, and overall quality of life, including cognitive status [1]. Non-effective pain management can cause various complications, including ileus, nausea, prolonged hospital stay, poor surgical outcome, and chronic pain syndromes [2]. Opioids, which have been mainly used for treating chronic spinal pain, are commonly used as first-line agents of perioperative analgesia [3]. However, the chronic use of this therapy seems to be associated with hyperalgesia and postoperative pain, due to peripheral and central sensitisation, cognitive impairment, and respiratory depression [4]. A multimodal approach incorporating both opioid and nonopioid agents has been effectively employed to mitigate postoperative pain and reduce opioid-related side effects [5]. Nonopioid anaesthesia methods may help eliminate iatrogenic postoperative pain and the need for opioid analgesia [1].

The use of α2 adrenergic receptor agonists (dexmedetomidine (DEX) and clonidine) can contribute to haemodynamic stability and a variety of therapeutic effects [6]. DEX is a highly selective α2-adrenergic receptor agonist, exhibiting seven to eight times greater selectivity than clonidine [7]. The activation of these receptors, located in the central venous system and the spinal cord, leads to the inhibition of adenylyl cyclase, which catalyses cAMP (Cyclic AMP) formation, an important second messenger in various cellular catabolic pathways. Moreover, alterations in cellular ion conductance and membrane hyperpolarisation diminish noradrenergic activity, stress responses and sympathetic outflow, resulting in improved myocardial oxygenation and blood supply, and enhanced recovery quality [8,9]. DEX exhibits anxiolytic, sedative, anti-inflammatory, and analgesic effects, while also promoting respiratory and haemodynamic stability. Exceptions include patients with volume depletion, severe heart block, or vasoconstriction, in whom DEX should be used cautiously due to the risks of bradycardia and hypotension [7]. Ultimately, when combined with commonly used analgesics, DEX may enhance postoperative pain control and improve overall quality of life.

Postoperative cognitive dysfunction (POCD), a common perioperative neurological complication in the elderly, is characterised by impairments in language, memory, task execution, and perceptual functions. In contrast, postoperative delirium (POD) typically presents in the early postoperative period and is marked by disorientation, purposeless movements, and restlessness. POD results from acute cerebral dysfunction and is defined by disturbances in attention, awareness, and cognition [10]. This type of delirium, presented in patients undergoing spinal surgery, is reported with an incidence up to 40.5% [11]. DEX has been shown to improve N3 sleep, leading to the enhancement of neurocognitive function and a reduction in postoperative delirium [12]. Moreover, the impact of DEX on postoperative pain management is reported to affect cognition, as sleep disorders on the first postoperative night, often caused by severe pain, can be predictive factors for delirium [13]. Consequently, DEX appears to be effective in maintaining and improving neurocognitive function and reducing the risk of postoperative delirium.

Although dexmedetomidine is an effective alternative to opioid analgesics used in anaesthesia care and is associated with fewer opioid-related complications, its integration into anaesthetic practice remains limited. According to the literature, its limited intraoperative use is due to a lack of clinical experience and knowledge [14].

The aim of this review is to enhance current understanding and encourage further research into the beneficial effects of perioperative DEX on pain management and cognitive recovery following spinal surgery.

## 2. Materials and Methods

### 2.1. Literature Research

**Figure 1:**
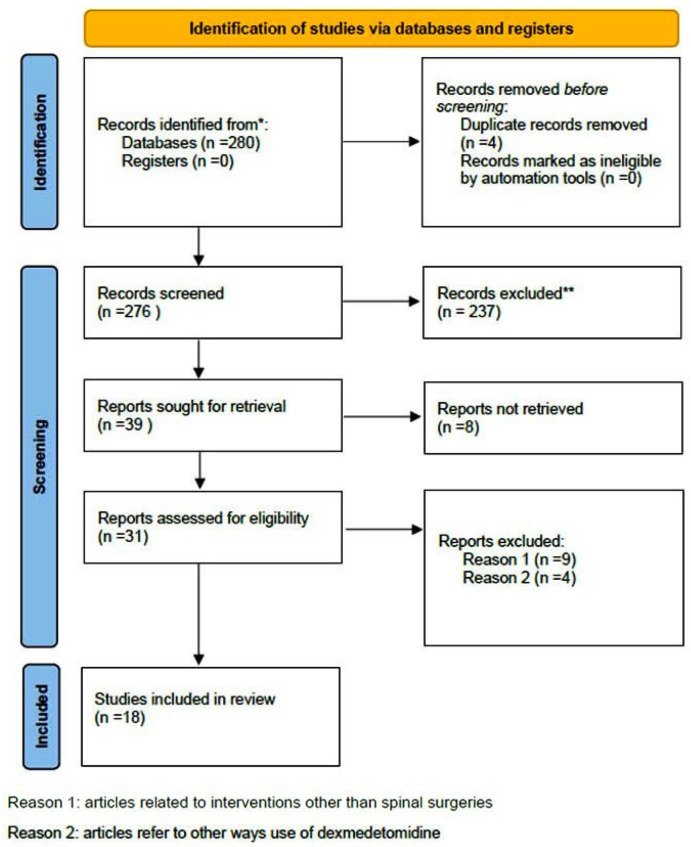
PRISMA 2020 flow diagram for new systematic reviews which included searches of databases and registers regarding pain management. (* Records identified in both databases (Pubmed and Web of Science), ** Records excluded by the authors without automation tools).

**Figure 2:**
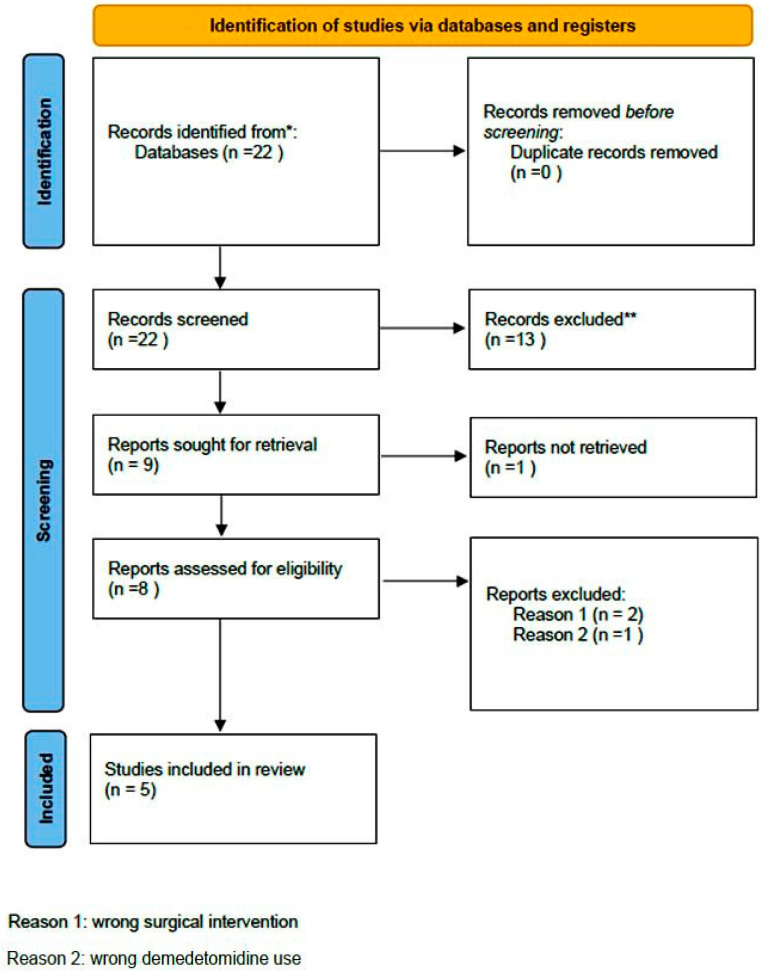
PRISMA 2020 flow diagram for new systematic reviews which included searches of databases and registers regarding delirium. (* Records identified in both databases (Pubmed and Web of Science, ** Records excluded by the authors without automation tools).

A comprehensive computer-based literature search was conducted on 02/05/2025 to investigate the use of dexmedetomidine (DEX) in relation to postoperative pain control, cognitive function, and spinal surgery. The databases searched were Web of Science (1900 to present) and PubMed (1947 to present). The search methodology used a combination of the terms “dexmedetomidine [All Fields]”, “spinal surgery [All Fields]”, “pain control [All Fields]”, and “delirium” [All Fields]. Duplicate records were removed using Zotero version 6.0.10 (Digital Scholar, https://digitalscholar.org/, accessed on 20 April 2025 ). The systematic review was conducted according to PRISMA guidelines and is presented in the following PRISMA flow charts (Figure 1 and Figure 2). Moreover, the study was registered in the PROSPERO (International Prospective Register of Systematic Reviews) database with registration number CRD420251082437, and full access to the protocol is provided.

### 2.2. Selection Criteria

The initial search included only studies published in English. All qualitative studies relevant to the main topics were retrieved. Additional inclusion criteria included (a) only full-text articles, (b) comparative studies assessing the systematic (intravenous) perioperative use of dexmedetomidine and other anaesthetics in all types of spinal surgeries in adults (aged over 18 years), focusing on pain control and cognitive function, and (c) studies evaluating outcomes of perioperative dexmedetomidine administration in spinal procedures. No restrictions were placed on the date of publication. Exclusion criteria were (a) studies based on in vitro or in vivo animal models, (b) case reports, case series, letters to the editor, or studies with insufficient data regarding the period of use and clinical outcomes, (c) studies focusing on wound infiltration with dexmedetomidine rather than its systematic administration, and (d) studies written in languages other than English.

### 2.3. Data Extraction

The literature research and data extraction were conducted independently by two authors (S.R.A. and I.L.) and an experienced librarian. The authors screened titles and abstracts in accordance with the inclusion and exclusion criteria and identified articles relevant to the favourable effects of DEX on perioperative or postoperative analgesia and cognitive function or delirium. Disagreements regarding inclusion were resolved by the senior author (S.G.P.). Relevant data were then extracted and recorded in a Microsoft Excel spreadsheet (Microsoft Office 365, Redmond, WA). Any disagreements between the two authors on the data extracted were also resolved by the senior author after the re-evaluation of the full texts.

This study adheres to the principles of systematic reviews, aiming to synthesise the existing literature conscientiously.

### 2.4. Evaluation Analysis

The methodology of each original study was assessed independently by two authors (A.K. and R.S.A.) using the Newcastle–Ottawa quality assessment scale [15]. Included studies were graded using a three-category scale. Studies displaying a total score of 0–3, 4–6, and 7–9 were classified as poor-, fair-, or good-quality, respectively. Review articles were not evaluated by the above assessment scale.

## 3. Results

### 3.1. Included Studies

A total of 21 studies met the inclusion criteria and are presented in two tables: Table 1 [16,17,18,19,20], and Table 2 [17,21,22,23,24,25,26,27,28,29,30,31,32,33,34,35,36]. Three of these were review articles [25,26,35].

### 3.2. Quality Assessment

According to the Newcastle–Ottawa scale, all included trials were considered of high quality and were therefore deemed to be at a low risk of bias (Table 1 and Table 2).

## 4. Discussion

### 4.1. Dexmedetomidine and Anti-Inflammatory Activity in Pain Control

The wide range of spinal surgeries performed today presents significant challenges for anaesthetic teams aiming to maintain perioperative haemodynamic and respiratory stability, achieve effective pain control, and facilitate recovery. Surgery and anaesthesia stress combined with excessive bleeding cause an inflammatory response, which leads to impaired body function and side effects [35]. Several drugs, including dexmedetomidine, have been used in order to suppress this response.

DΕΧ acts at the level of the spinal cord as a highly selective a2 adrenergic receptor agonist, which primarily inhibits signal transmission in the dorsal horns by activating pre-synaptic a2 adrenergic receptors. The activation of this receptor leads to internal calcium ion flow, resulting in decreased neurotransmitter release and the inhibition of signal transduction from peripheral nerve fibres [37]. Moreover, findings from animal experiments suggest that DEX can activate anti-apoptotic agents and thus protect cells, by decreasing CD14+ and CD42a+ (T2, T3, and T4 cells) and increasing HLDRA+ and CD14+ (T4 and T5 cells) [38]. The changes in the concentration of this agent contribute to reducing lipid peroxidation and ischemia–reperfusion injury and suppressing the inflammatory response [39].

Animal studies have shown that DEX can block the transmission of neural signals from peripheral neurons and thus contribute to perioperative pain control [36]. Its short duration of action and plasma half-life (approximately 2.3 h) compared to clonidine make it an effective adjuvant for immediate postoperative analgesia. The short- and long-term action of DEX has been widely studied in clinical research [18,20,21,22,23,24,25,26,27,29,30,31,32,34,35].

Hwang et al. [31] reported the superior analgesic efficacy of DEX compared with remifentanil for up to 48 h postoperatively. These results are attributed to its action on nociceptors, located in laminae I-III of the dorsal horns, where it modulates pain thresholds, resulting in improved postoperative pain management. Similarly, when the combined use of DEX and fentanyl (first group) was compared to the use of fentanyl plus droperidol (second group), a lower need for intraoperative fentanyl was reported in the first group, indicating greater efficacy compared to other anaesthetics [25]. Rahimzadeh et al. also noted decreased perioperative and postoperative analgesic use when DEX was used [30]. Moreover, patients receiving perioperative DEX required supplemental analgesia later than those receiving fentanyl [21]. Bojaaraj et al. reported better pain scores at 1 and 2 h postoperatively in patients undergoing elective spine surgery with DEX compared with controls receiving saline [32]. Results on a more extended postoperative period were reported in a study by Nikhoubakht et al. [24], who revealed a statistically significant difference regarding the use of opioids between dexmedetomidine and the control group after posterior spinal fusion until 24 h following surgery. Similarly, Kim et al. extended the postoperative follow-up to 48 h and observed better pain scores in the DEX group at 1 h postoperatively. However, no statistically significant difference in the long-term results was reported between the two groups (1–6 h, 6–24 h, and 24–48 h postoperatively) [22].

Results from other studies regarding the impact of dexmedetomidine on postoperative pain perception and requirements for opioid analgesics remain inconclusive, as no statistically significant outcomes were revealed in various clinical cases [18,24,26,28,29,34,35]. The different duration of the follow-up periods for postoperative pain evaluation (after drug withdrawal), under 48 h in most cases, resulted in heterogeneity and incomplete data on the long-term response to DEX administration [24,28,35].

Differences in surgical settings the drugs used may also explain inconsistencies in the findings, as reported by Choi et al., where the comparable efficacy of dexmedetomidine and remifentanil within 48 h postoperatively was observed [28]. Even though a lower opioid consumption was noted in patients receiving dexmedetomidine, Naik et al. did not find statistically significant differences in postoperative opioid use or pain scores during a three-day follow-up after major spinal surgery [29]. This effect may be attributed to the perioperative use of methadone, a long-acting, opioid-sparing analgesic, which could have masked the action of DEX. Similarly, the opioid-sparing effect of DEX on pain management could be masked by the different ketorolac consumption and analgesics mix, through patient-controlled intravenous anaesthesia, as seen in the clinical outcomes reported by Song et al. [34].

The diversity in DEX dosages used can affect the results reported in some studies [24]. The comparison between groups under ketamine or DEX revealed statistically significant differences in results in the immediate postoperative period (less than 24 h), regarding the need for additional analgesia with opioids, but no significant differences in pain control. These results may reflect the unusually relative low dose of dexmedetomidine used in this study [24]. Even in studies with longer follow-up periods (up to five days), small sample sizes and varied subjective pain assessment tools contributed to a lack of statistically significant findings in postoperative pain scores. According to Zhou et al. [35], although dexmedetomidine can limit immunosuppression and the inflammatory response, postoperative pain does not appear to affect sympathetic response [35]. In contrast with other studies, Garg et al. reported better pain scores in the group treated with ketamine compared with those under DEX, although both groups outperformed the control group [33].

According to the existing literature reviewed by Tsaousi et al. and Waelkens et al., over the past years, DEX has emerged as an efficient opioid-sparing agent during the intraoperative period, but its postoperative efficacy remains unclear [26,36]. The lack of consensus on the results of dexmedetomidine on pain management and opioid consumption following spinal surgeries could be explained by the heterogeneity of the parameters of these clinical trials. DEX could be combined with various other pharmacological agents, used in different dosages and models of drug administration. Moreover, inconsistencies in subjective pain scoring systems, the usually short-term follow-up periods, and opioid-induced hyperalgesia limit the interpretation of its analgesic role to a short postoperative period (up to 6 h) [36].

### 4.2. Impact of Dexmedetomidine on Postoperative Cognitive Function and Delirium

Postoperative cognitive dysfunction (POCD) is characterised by impaired task assembly ability, memory, perceptual or language functions and differs from postoperative delirium (POD), which mainly presents with disturbance in memory, attention, awareness, cognition, and consciousness. Postoperative delirium presents with three subtypes: hypoactive, hyperactive, and mixed [40]. Both POCD and POD are common perioperative and postoperative neurological disorders [41]. Their notable association can be explained by the elevation of pro-inflammatory cytokines in the plasma and cerebrospinal fluid of elderly patients presenting with delirium post-surgically (first postoperative day). The normalisation of their trend on the third postoperative day often coincides with the resolution of delirium symptoms.

According to Ye et al., DEX may modulate the immune response and reduce postoperative delirium occurrence, by mitigating the early elevation of IL-6 and TNF-α. This agent inhibits signals related with inflammation and cell death (the downregulation of phospho-JNK-phosphorylated c-Jun N-terminal kinase) and enhances neuroprotection pathways (the upregulation of phosphor-ERK1/2-phosphorylated extracellular signal regulated kinase 1/2 expression) [20]. Niu et al. reported superior outcomes on postoperative delirium with intratracheal or intravenous DEX compared to intranasal use [16]. Intravenous use was also associated with better sleeping patterns. Similarly, DEX appears to reduce the incidence of hyperactive postoperative delirium in patients undergoing spine surgery for lumbar degenerative diseases [19].

The efficacy of DEX combined with esketamine on POCD in patients treated with elective lumbar spine surgery was studied by Tao et al. [18]. The combination of these drugs can be more effective in reduction in POCD incidence on the first postoperative day than either agent alone. No statistically significant difference was observed between the esketamine–DEX group and the DEX-only group. However, these results could be influenced by the relatively low presence of POCD in these patients, due to the advanced surgical techniques that reduce trauma effect. Moreover, pain scoring scales presented better results in the group of patients under esketamine and DEX at 2 and 24 h postoperatively, which is in line with results regarding the incidence of POCD, as this possibly indicates their role in the suppression of inflammation and neuroprotection [37]. Finally, various side effects, including emergence agitation or delirium, which are presented with disorientation, uncontrolled movements, and excitation during early aesthesia recovery, could be avoided with a well-controlled dosage of DEX, which reduces haemodynamic adverse events, especially in older patients (aged over 65 years) [17].

### 4.3. Dexmedeomidine, Pain Management, and Delirium

Postoperative pain is a recognised risk factor for the development of POD [40]. However, pain assessment scales were incorporated in only two of the studies focusing on POD or POCD [18,20]. Although DEX has been shown to significantly reduce the incidence of POD and attenuate short-term elevations in IL-1, IL-6, and TNF-α, no statistically significant differences in pain scores were observed between patients receiving DEX and those who did not, according to Ye et al. [20]. These results derive from a single-centre study conducted in a secondary hospital, which suffered from considerable patient loss during a relatively short follow-up period, resulting in incomplete pain assessment data that may have influenced the outcomes. Similarly, Tao et al. studied the effect of esketamine combined with DEX on VAS (visual analogue scale) scores, in addition to its impact on POCD. The combination of these drugs revealed superior outcomes on pain control at 2 and 24 h than each drug alone, but not at the long-term follow-up (48 h) [18]. Further studies are warranted to clarify the direct relationship between perioperative and postoperative pain control, inflammatory processes, and cognitive function. These investigations should aim to determine the optimal DEX dosage for achieving both analgesic and neuroprotective effects.

## 5. Strengths and Limitations

To the best of our knowledge, this review represents the most up-to-date synthesis on the analgesic effect of DEX, incorporating the latest clinical trial data on this issue and is the only one focusing on its outcomes both on pain management and postoperative cognitive dysfunction or delirium. However, our study has several limitations. Although 21 entries of high quality were included in this review, the studies’ designs and methods were heterogeneous as different clinical protocols were used and no standardised methods were applied in order to evaluate the reproducibility of the outcomes. Moreover, the limited experience in clinical settings raises concerns about the long-term results of DEX infusion in patients undergoing spinal surgeries. Finally, a language bias could be present as only studies written in English were reviewed.

## 6. Conclusions

Recent studies have explored the pain-relieving and neuroprotective effects of DEX in spinal surgery. However, the heterogeneity of dosing protocols, surgical procedures, and the tools used to assess pain and cognitive outcomes has led to discrepancies in findings and a lack of statistically significant results across several trials. Moreover, the association between pain control, inflammation response, and delirium or postoperative cognitive dysfunction remains insufficiently studied. The present review study supports the intraoperative administration of dexmedetomidine in decreasing the pain intensity and/or opioid consumption as well as the development of POD and POCD in haemodynamically stable patients undergoing spinal surgeries during the first 24 h postoperatively. The outcomes of this study should be interpreted with caution and should not yet be generalised. Future studies on different parameters of dexmedetomidine administration and its impact on pain management and cognitive function over longer follow-up periods could contribute to more efficient and safe outcomes.

## Figures and Tables

**Table 1:** The literature on the intraoperative and postoperative role of DEX in pain management.

Study	Type of Study	Number of Patients/Number of Studies	Type of Surgery	Purpose of Study	Newcastle–Ottawa Score	Results	Comments
Turgut et al., 2008 [21]	Clinical article	50	Lumbar laminectomy	Compare fentanyl/DEX * on perioperative haemodynamic conditions, propofol need, and side effects	08 (Good)	Earlier need for analgesics in fentanyl group (median time 35 min vs. 60 min); greater incidence of vomiting and nausea in the same group	DEX * offers more rapid recovery and reduced need for pain medication, thus reducing total length of stay
Naik et al., 2014 [29]	Clinical article	142	>3 level of thoracic/lumbar spine deformity surgery	Evaluate impact of DEX * on postoperative pain management and need for opioids	09 (Good)	Lower intraoperative opioid consumption, but not statistically significantly lower postoperative consumption or improved pain scores (up to 3 days)	Methadone mask beneficial effect of DEX *
Rahimzadeh et al., 2014 [30]	Original article	60	Posterior spinal fusion	Compare remifentanil with DEX * in spinal surgery	08 (Good)	Lower intraoperative and especially postoperative consumption of analgesics	Single-centre study with small population
Hwang et al., 2015 [31]	Clinical research article	40	Posterior lumbar interbody fusion	Compare remifentanil with DEX * on postoperative need for analgesics	08 (Good)	VAS ** score of remifentanil group significantly greater than DEX * one, every time following surgery (*p* < 0.05)	DEX * more efficient in pain control at first 48 h, but effective for extended period, too
Bojaraaj et al., 2016 [32]	Original article	60	Elective spine surgery	Evaluate efficacy and safety of intravenous DEX * on haemodynamic changes and anaesthetic challenges	07 (Good)	Significant lower amount of propofol in the dexmedetomidine group intraoperatively, to maintain haemodynamics	Statistically significantly better pain scores in dexmedetomidine group at 1 h and 2 h postoperative (*p* = 0.0001)
Garg et al., 2016 [33]	Clinical research article	66	Elective spine surgery	Compare safety of opioid sparing analgesics	08 (Good)	Pain score statistically significantly lower in drug groups (except 0 h) and lower in ketamine group	Comparable results between ketamine and dexmedetomidine group (except 6 h postoperatively)
Song et al., 2016 [34]	Original article	108	Posterior lumbar spine fusion (one of two levels)	Compare impact of DEX * added to a fentanyl-based patient-controlled mixture on nausea (postoperative) and analgesia	09 (Good)	DEX * with reduced risk of postoperative nausea at first 1 to 3 h; consumption of fentanyl at each time greater in control group up to 6 h; total amount of pethidine during 48 h also greater	VAS ** scores without significant difference between groups; lower use of opioids in DEX * group
Zhou et al., 2017 [35]	Clinical research article	48	Multilevel fusion	Assess impact of DEX * on CD42a +, CD14+, and HLADR +/CD14+ levels and inflammatory cytokines	08 (Good)	No significant difference in VAS ** scores post-surgery. T2, T3, T4, T5, WBC, and CRP decreased significantly in DEX * group, with IL-6 and TNF-a markedly reduced.	Pain not related with inflammation level in both groups
Suzuki et al., 2018 [25]	Clinical article	45	Minimal invasive epiduroscopy	Compare conventional anaesthetics with DEX *	08 (Good)	Use of DEX * limits need for fentanyl compared with groups under fentanyl and dropiridol	Avoiding high doses of fentanyl with the use of DEX * can prevent respiratory depression or aspiration of elderly
Tsaousi et al., 2016 [36]	Review article	15 (studies)	Emergency or elective spinal surgery	Evaluate efficacy and safety of dexmedetomidine as sedative and analgesic agent in spine surgery	-	More efficient on intraoperative action when compared with placebo	Heterogeneity of clinical trials means that postoperative efficacy should be interpreted with extreme caution
Kim et al., 2019 [22]	Original article	52	Spine fusion Surgery	Impact of DEX * on stress responses	08 (Good)	Lower pain score at 1 h postoperatively	Similar pain scores at 1–6 h, 6–24 h, and 24–48 h postoperatively
Janatmankan et al., 2021 [23]	Research article	60	Lumbar discectomy	Compare DEX * and remifentanil regarding pain control	08 (Good)	Significant lower pain levels in DEX * group.	More stable haemodynamic conditions in the same group
Nikoubakht et al., 2021 [24]	Original article	87	Posterior spine fusion	Postoperative pain control in ketamine versus DEX * group	08 (Good)	Difference (significant) between groups regarding opioids prescribed during recovery and at 2, 4, 6, 12, and 24 h after surgery (*p* < 0.05)	No significant differences in ketamine and DEX * groups regarding pain intensity (low dose of DEX *)
Waelkens et al., 2021 [26]	Review article	35 (studies)	Complex spine surgery	Evaluate known research and recommendations regarding pain control	-	Lower need for perioperative opioids; better postoperative pain scores with DEX *	Limited procedure specific evidence on the use of intravenous DEX *; lower incidence of side effects (nausea, bradycardia, hypotension, and shivering)
Koh and Leslie, 2022 [27]	Review article	Not applicable	Complex spine surgery	Review research regarding agents and techniques for pain control	-	Emerging evidence for DEX * use for pain control and lower opioid requirement	No differences regarding hypotension and bradycardia; need for multimodal analgesia
Choi et al., 2023 [28]	Clinical trial	98	Lumbar spine surgery	Evaluate DEX * and remifentanil regarding postoperative sore throat	08 (Good)	Comparable results in pain scores and need for analgesics in both groups (1, 6, and 24 h postoperatively).	Results explained due to different surgical settings (time both agents stopped)
Tao et al., 2024 [18]	Clinical research article	162	Lumbar spine surgery	Evaluate of esketamine and DEX * on postoperative cognitive dysfunction	09 (Good)	VAS ** scores at 2 and 24 h significantly lower in esketamine DEX * group (*p* < 0.05); no significant differences in scores at 48 h postoperatively (*p* > 0.05)	Combination of drugs more effective in postoperative pain management
Ye et al., 2024 [20]	Clinical trial	218	Thoracolumbar compression fracture surgery	Assess influence of DEX * on delirium following surgery and inflammatory biomarkers	09 (Good)	Similar pain assessment scores between DEX * and control group at first (*p* = 0.748), second (*p* = 0.862), and third (*p* = 0.509); no statistically significant differences at these time points (*p* values > 0.05)	DEX * significantly mitigates short term elevation of IL-6 and TNF-α levels, contributes to short-term recovery in elderly patients; short term follow-up (three days)

* DEX: dexmedetomidine; ** VAS: visual analogue scale.

**Table 2:** The literature on the role of DEX on cognitive function. (* dexmedetomidine, ** postoperative delirium, *** postoperative cognitive dysfunction).

Study	Type of Study	Number of Patients	Type of Surgery	Purpose of Study	Newcastle–Ottawa Score	Results	Comments
Niu et al., 2023 [16]	Clinical research article	150	Elective spinal surgery	Study efficacy of administration routes of DEX * on POD **	09 (Good)	Incidence of POD ** in intravenous group significantly lower than in intranasal within 3 days (3 of 49 [6.1%] vs. 14 of 50 [28.0%]; *p* < 0.017); no difference between intratracheal and intravenous groups (5 of 49 [10.2%] vs. 3 of 49 [6.1%]; OR, 1.74; 95% CI, 0.40–7.73; *p* > 0.017)	Better sleep quality postoperatively in intravenous group; mild adverse events in all three groups
Ahn et al., 2024 [17]	Clinical article	44	Spine surgery (type not applicable)	Determine effective dose of DEX * intraoperative infusion to prevent emergence agitation	07 (Good)	At admission at the post-anaesthesia unit: one patient anxious, agitated, or restless; one with signs of excitement (quickly returned to tranquil state)	Effectiveness of DEX * on emergence agitation; stable recovery
Tao et al., 2024 [18]	Clinical research article	162	Lumbar spine surgery	Evaluate of esketamine and DEX * on POCD ***	09 (Good)	Incidence of POCD ** on first postoperative day significantly lower in esketamine–DEX * group compared to esketamine but not statistically different for DEX * alone compared with combination; on third postoperative day, differences between groups not significant	Combination eliminated serologic markers compared to individual drugs, revealing effective reduction in biomarkers of neuro inflammation and neuronal damage
Xie et al., 2024 [19]	Clinical article	7250	Lumber surgical treatment/degenerative diseases	Search changes in incidence of delirium in patients with lumbar degenerative disease and potential causes of changes	09 (Good)	Delirium related to age, number of elements, duration of surgical, remifentanil, benzodiazepines, and DEX * (*p* < 0.05)	Increase in dosage of DEX * reduces risk of delirium
Ye et al., 2024 [20]	Clinical trial	218	Thoracolumbar compression fracture surgery	Assess influence of DEX * on delirium following surgery	08 (Good)	On first postoperative day, DEX * group with significant reduction in POD ** compared with control group; overall incidence of POD lower in DEX * group; no significant differences in POD ** on second and third postoperative days	DEX * mitigates short-term elevation of IL-6 and TNF-α levels, and contributes to short-term recovery in elderly patients
